# Gastric Duplication: A Rare Cause of Recurrent Vomiting

**DOI:** 10.1155/2017/2348274

**Published:** 2017-03-01

**Authors:** Brahmananda Koduri, Katie McHale, Christina Yost, Michael H. Goodman, Dennis Hoelzer

**Affiliations:** Department of Pediatrics, The Children's Regional Hospital, Cooper Hospital-University Medical Center, Camden, NJ 08103, USA

## Abstract

Vomiting is a physical finding that can occur at any age but presents the greatest challenge when it is recurrent in a child. The etiology is varied (Sieunarine and Manmohansingh, 1989; Suzuki, 1982), and recurrent vomiting can be a symptom of life threatening medical or surgical emergencies. Early recognition is mandatory for preventing delay in management and potential complications. Gastric duplication is rare and mostly diagnosed in infancy with only a few cases documented in the medical literature presenting in childhood. We present a three-year-old Vietnamese female with recurrent vomiting. Obstruction and sepsis were ruled out as a cause of the recurrent vomiting by history and appropriate tests. Persistent vomiting and paucity of air on the plain abdominal films provided a clue to the diagnosis. A CT scan of the abdomen with contrast revealed a uniformly thin walled fluid attenuation mass in the epigastric region which did not opacify with contrast. An abdominal ultrasound confirmed gastric duplication cyst and the patient was taken to the operating room for excision of the cyst.

## 1. Introduction

Vomiting is a presenting complaint in a multitude of disorders (Table 1) [1, 2] ranging from gastrointestinal pathology, increased intracranial pressure, and disease in distant organs including the brain. The ability to vomit presumably conveys a survival advantage by enabling the expulsion of toxins from the stomach. Vomiting occurs after stimulation of either the vomiting center (VC), a central “control center” in the medulla near the respiratory center, or the chemoreceptor trigger zone (CTZ) in the area postrema on the floor of the fourth ventricle. Recurrent vomiting is at least 3 episodes over a 3-month period while cyclic vomiting is recurrent, intense episodes separated by asymptomatic periods.

## 2. Case Report

A 3-year-old Vietnamese female was transferred from an outlying Emergency Department for intractable nonprojectile vomiting. She was born by full term spontaneous vaginal delivery with no perinatal complications. The patient had no significant past medical problems and did not take any medication on a regular basis. Vomiting started 2 days prior to admission with each meal. On the day of admission the patient experienced approximately 30 episodes of vomiting which were nonbloody and nonbilious. She also developed nonradiating abdominal pain in the epigastric region. The pain, described as 10/10 by faces charts, was episodic in nature with some periods of relief. No aggravating or relieving factors were noted. There was no diarrhea. The child had decreased urine output, decreased appetite, and increased thirst, along with weakness and fatigue. Her last bowel movement was three days prior to admission. The patient never had any history of constipation, and she had regular bowel movements prior to this episode. The family history was significant for asthma in her father and no migraines. Social history was significant for no pets or travel. Her father smokes. There was no history of substance abuse. There were no reported familial or social stressors.

Laboratory results from the outlying Emergency Department showed a normal CBC and BMP. A UA revealed ketones, 5–10 WBCs, and trace protein. LFTs showed a slightly elevated amylase of 162. The patient was admitted for dehydration and evaluation of her abdominal pain and persistent vomiting.

On physical exam, weight 13.6 kg (10–25th percentile), height 95 cm (10th percentile), and BMI 15 (25th percentile). Vital signs were *T* 99.3°F, HR 131, RR 26, BP 84/47, and oxygen saturation 97% on RA. Skin turgor was normal. The patient had dry skin and lips, her eyes were not sunken, and tears were present with crying. The GI exam revealed no tenderness on superficial palpation. On deep palpation there was generalized tenderness. There was no guarding, rigidity, rebound tenderness, hepatosplenomegaly, or masses. Bowel sounds were normal. She had minimal right sided costovertebral angle tenderness. The rectum had normal tone and occult blood test was negative.

Blood and urine cultures were obtained and she was started on cefotaxime for empiric coverage for urinary tract infection, despite being afebrile. The patient remained NPO and was started on maintenance IV fluids. The patient's diet was advanced to clear liquid after vomiting had ceased for a few hours. Immediately after drinking clear liquid the patient vomited and was once again made NPO.

**Figure 1:**
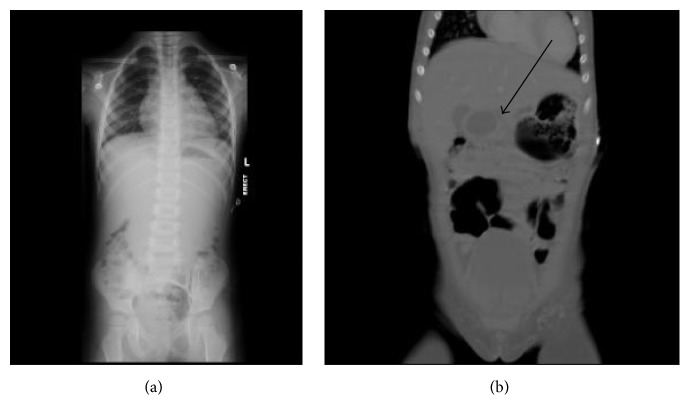
(a) Obstruction series. (b) CT with PO and IV contrast.

An obstruction series at the outlying Emergency Department was read as negative for obstruction. A repeat 3-view obstruction series (Figure 1(a)) was obtained after admission because of continued vomiting. The repeat obstruction series was negative for obstruction, but there was a small right pleural effusion, ascites, and a paucity of gas in the epigastric region, which suggested a mass. A CT with PO and IV contrast of the abdomen was obtained. The patient had difficulty tolerating the PO contrast but consumed half of the required amount. The CT scan revealed a uniformly thin walled fluid attenuation mass in the epigastric region, which did not opacify with contrast, suggesting a duplication cyst or a choledochal cyst (see arrow in Figure 1(b)). Correlation with ultrasound was recommended, to evaluate “gut signature” or continuity with the biliary tract. If this was inconclusive, MRI with MRCP was suggested. An abdominal ultrasound revealed a 2–6 cm cystic structure in the epigastric region suggestive of enteric duplication cyst, but a choledochal mass could not be excluded.

**Figure 2:**
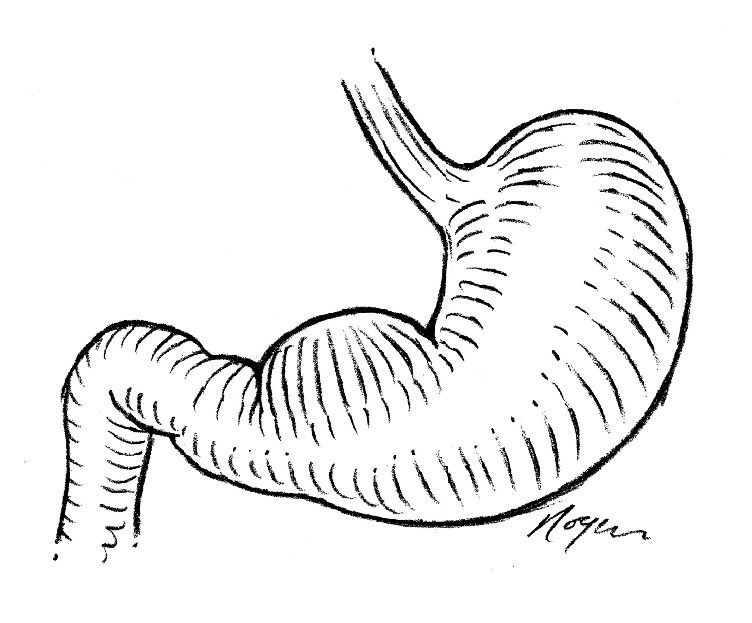
Cystic duplication sketch. Sketch by Paul Rogers, CMI, medical illustrator/photographer/videographer/graphics, Cooper University Hospital.

On day 2 of admission a surgical consult was obtained. The child remained NPO with an NG tube. A GI consult recommended starting PPI, Toradol for abdominal pain, and an MRI with MRCP. On day 3 of admission, the patient had an episode of hematemesis and was taken to surgery. A transverse incision running from the midline just above the umbilicus to the anterior axillary line was used to enter the abdomen. A cystic duplication, measuring approximately 5 cm in width, was identified over the distal greater curvature of the stomach (Figure 2).

The duplication was opened and hemorrhagic secretions were observed. The common wall of the cyst and the stomach was wedge resected. The appendix was visualized and removed using electrocautery for hemostasis along with two stick ties of 3-0 Ethibond suture to secure the stump of the appendix. The stomach was closed in a 2-layer fashion using a running suture of 3-0 Vicryl to reapproximate the wall of the stomach. Lembert sutures of 3-0 Vicryl and an omental patch over the area were used to reinforce the area. The abdomen was closed in layers with suture and Steri-Strips and Mastisol was used as a dressing, covered with Tegaderm.

Postoperatively the patient remained NPO after surgery. She was started on a clear liquid diet when bowel sounds were appreciated. The diet was then advanced to a full diet as tolerated. The lipase had returned to normal postoperatively. The patient was discharged home on day 5 of admission, as she was tolerating a regular diet, with no complaints of abdominal pain and no vomiting.

The patient was seen for a post-op visit one week after discharge. The patient was tolerating her regular diet, free of abdominal pain, and not having any episodes of vomiting. The wound was clean, dry, and intact and Steri-Strips were removed.

Surgical pathology showed gastric duplication with hemorrhagic adhesive fibromembranous tissue externally, 3.6 × 2.5 cm and 0.5 cm in thickness. There was gastric type mucosa with mural focus of pancreatic tissue. Appendix was without pathology.

## 3. Discussion

Vomiting for days with each meal in an otherwise healthy 3-year-old patient is frequently seen in gastroenteritis (GE). GE is usually associated with diarrhea and 30 episodes of vomiting is not usual. Localized abdominal pain in the epigastric region without a palpable mass often can be seen in acute gastritis and gastroesophageal reflux disease. These combination of symptoms can also be noted in systemic infections like urinary tract infection or abdominal migraine. Cyclic vomiting may present in young children as an early precursor of migraine.

A surgical condition such as a gastric duplication cyst usually presents at younger than one year of age with vomiting, poor feeding, failure to gain weight, and a palpable mass upon physical examination. All these features described were not present in our patient. The mucosal lining of the gastric duplication cyst is often gastric [3] and can lead to melena or hematemesis, which manifested late in our patient. Recurrent pancreatitis may occur from gastric duplications communicating with the pancreatic duct [4, 5]. Our patient initially had an elevated lipase which resolved postoperatively, indicating an acute pancreatitis. Gastric duplications are generally cystic [2], located on the greater curvature of the stomach, and have no communication to the stomach. In most cases, resection can be accomplished without entering the stomach by resecting the shared wall between the stomach and the duplication.

## 4. Conclusion

Patients presenting with recurrent vomiting with normal labs and cultures should have further testing. Ultrasound of the abdomen should be the next step in order to rule out any structural causes [6]. In our case, a CT scan with contrast of the abdomen aided in diagnosing a mass, although an ultrasound was needed to confirm gastric duplication.

## Figures and Tables

**Table 1:** Differential diagnosis of emesis during childhood.

Infant	Child	Adolescent
*Common*		
Gastroenteritis	Gastroenteritis	Gastroenteritis
Gastroesophageal reflux	Systemic infection	GERD
Overfeeding	Gastritis	Systemic infection
Anatomic obstruction	Toxic ingestion	Toxic ingestion
Systemic infection	Pertussis syndrome	Gastritis
Pertussis syndrome	Medication	Sinusitis
Otitis media	Reflux (GERD)	Inflammatory bowel disease
	Sinusitis	Appendicitis
	Otitis media	Migraine
		Pregnancy
		Medication
		Ipecac abuse/bulimia

*Rare*		
Adrenogenital syndrome	Reye's syndrome	Reye's syndrome
Inborn error of metabolism	Hepatitis	Hepatitis
Brain tumor (increased intracranial pressure)	Peptic ulcer	Peptic ulcer
	Pancreatitis	Pancreatitis
Subdural hemorrhage	Brain tumor	Brain tumor
Food poisoning	Increased intracranial pressure	Increased intracranial pressure
Rumination	Middle ear disease	Middle ear disease
Renal tubular acidosis	Chemotherapy	Chemotherapy
	Achalasia	Cyclic vomiting (migraine)
	Cyclic vomiting (migraine)	Biliary colic
	Esophageal stricture	Renal colic
	Duodenal hematoma	
	Inborn error of metabolism	
